# Successful Treatment of a Drug-Resistant Epilepsy by Long-term Transcranial Direct Current Stimulation: A Case Report

**DOI:** 10.3389/fneur.2018.00065

**Published:** 2018-02-09

**Authors:** Daniel San-Juan, Carlos Ignacio Sarmiento, Katia Márquez González, José Manuel Orenday Barraza

**Affiliations:** ^1^Department of Clinical Research, National Institute of Neurology and Neurosurgery, Mexico City, Mexico; ^2^Department of Basic Sciences and Engineering, Autonomous Metropolitan University Campus Iztapalapa, Mexico City, Mexico; ^3^Superior School of Medicine, National Polytechnic Institute, Mexico City, Mexico

**Keywords:** transcranial direct current stimulation, cortical dysplasia, neuromodulation, epilepsy, pharmaco-resistant

## Abstract

Transcranial direct current stimulation (tDCS) is a reemerged noninvasive cerebral therapy used to treat patients with epilepsy, including focal cortical dysplasia, with controversial results. We present a case of a 28-year-old female with left frontal cortical dysplasia refractory to antiepileptic drugs, characterized by 10–15 daily right tonic hemi-body seizures. The patient received a total of seven sessions of cathodal tDCS (2 mA, 30 min). The first three sessions were applied over three consecutive days, and the remaining four sessions of tDCS were given each at 2-week intervals. At the 1-year follow-up, the patient reported to have a single seizure per month and only mild adverse events.

## Introduction

Transcranial direct current stimulation (tDCS) is a noninvasive cerebral therapy, which has been tested in several neuropsychiatric conditions, including epilepsy, in recent years (1, 2). Anodal tDCS causes neuronal depolarization and increases excitability, while cathodal tDCS diminishes it (3, 4). Following this argument, cathodal tDCS has been proposed as a therapy to diminish the epileptic seizures and epileptiform interictal discharges in animal models and patients who have pharmaco-resistant epilepsy, or who are not candidates for epilepsy surgery, showing preliminary safety and efficacy (2, 5). However, the use of tDCS in patients with focal cortical dysplasia is controversial (6, 7).

Previous studies in patients with epilepsy using cathodal tDCS applied different stimulation parameters, ranging from 1 to 20 sessions for 20–60 min using 1–2 mA for 10–60 min (6–8), with 12 months of follow-up (8). Interestingly, the studies with higher number of tDCS sessions and long-term follow-ups are rare and have shown positive effects (7, 8). Our group previously published the use of cathodal tDCS in 20 patients with mesial temporal lobe epilepsy and hippocampal sclerosis with good results (−43.4 to −54.6%) in the reduction of seizures of three and five sessions during 60 days of follow-up (2). Then, we extended this treatment to other types of epilepsies as Rasmussen’s encephalitis (8) and other focal extra-temporal epilepsies, such as frontal lobe epilepsy. Here, we present an initial single case report from this series.

We aimed to depict a patient with frontal cortical dysplasia refractory to antiepileptic drugs (AEDs), who underwent seven sessions of cathodal tDCS over a period of 9 weeks and a long-term follow-up of 1 year with an improved epilepsy control. We have formerly obtained a written informed consent from the patient for the publication of this study case.

## Patient Case

**Figure 1:**
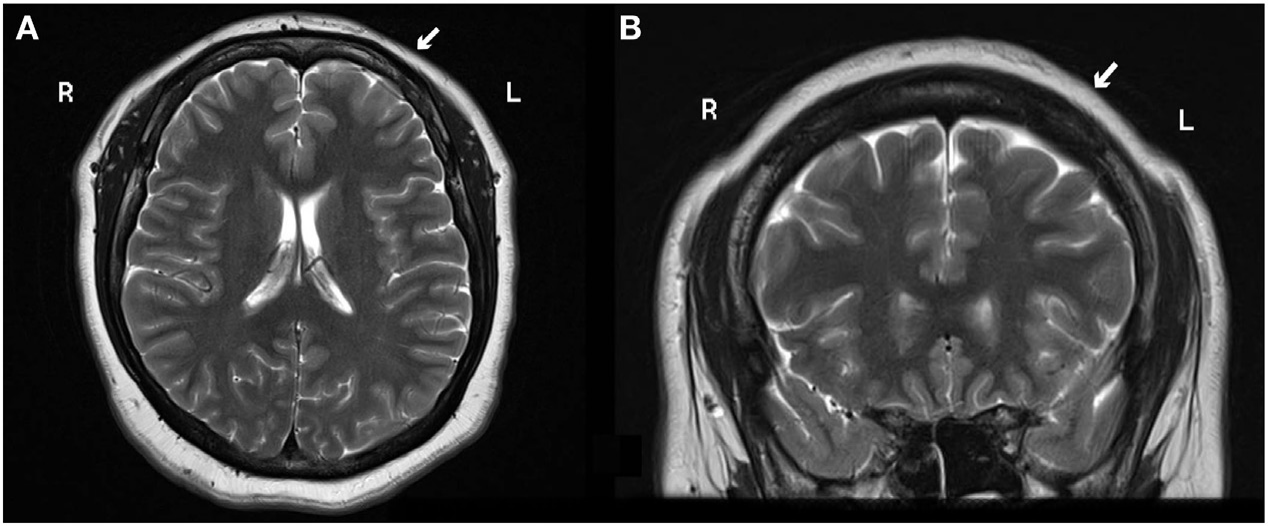
**(A)** Axial and (**B)** coronal 3 T MRI head scan shows blurry cortical gray and white matter on the left frontal lobe suggestive of focal cortical dysplasia (white arrow). Abbreviations: R, right; L, left.

A 28-year-old right-handed female presented to our service with neuroimaging findings compatible with frontal cortical dysplasia (Figure 1) refractory to AEDs. The patient had a normal neurodevelopment and her past medical history was noncontributory. She had her first seizure at 9 years old, described as a clonic seizure of right lower limb accompanied by nervousness and fear, with a duration of 30 s and sudden disappearance of symptoms, with a frequency of once a month. At 12 years of age, she had her first tonic-clonic seizure related to the onset of her menses. She was evaluated at 15 years old at our institution and was treated with carbamazepine showing good response. The patient was seizure-free for 5 years, but at the age of 20, her focal seizures increased in frequency to 1–2 times a month. These episodes of clonic and sensory right hemi-body seizures lasted for 3 years, and eventually worsened to 10–15 seizures per day. By that time, she was being medically treated with carbamazepine (200 mg/TID), valproic acid (600 mg/TID), and lamotrigine (100 mg/BID), with side effects consisting of somnolence, fatigue, and dizziness, and with serum levels of AEDs in therapeutic ranges. She denied any previous history of status epilepticus.

**Figure 2:**
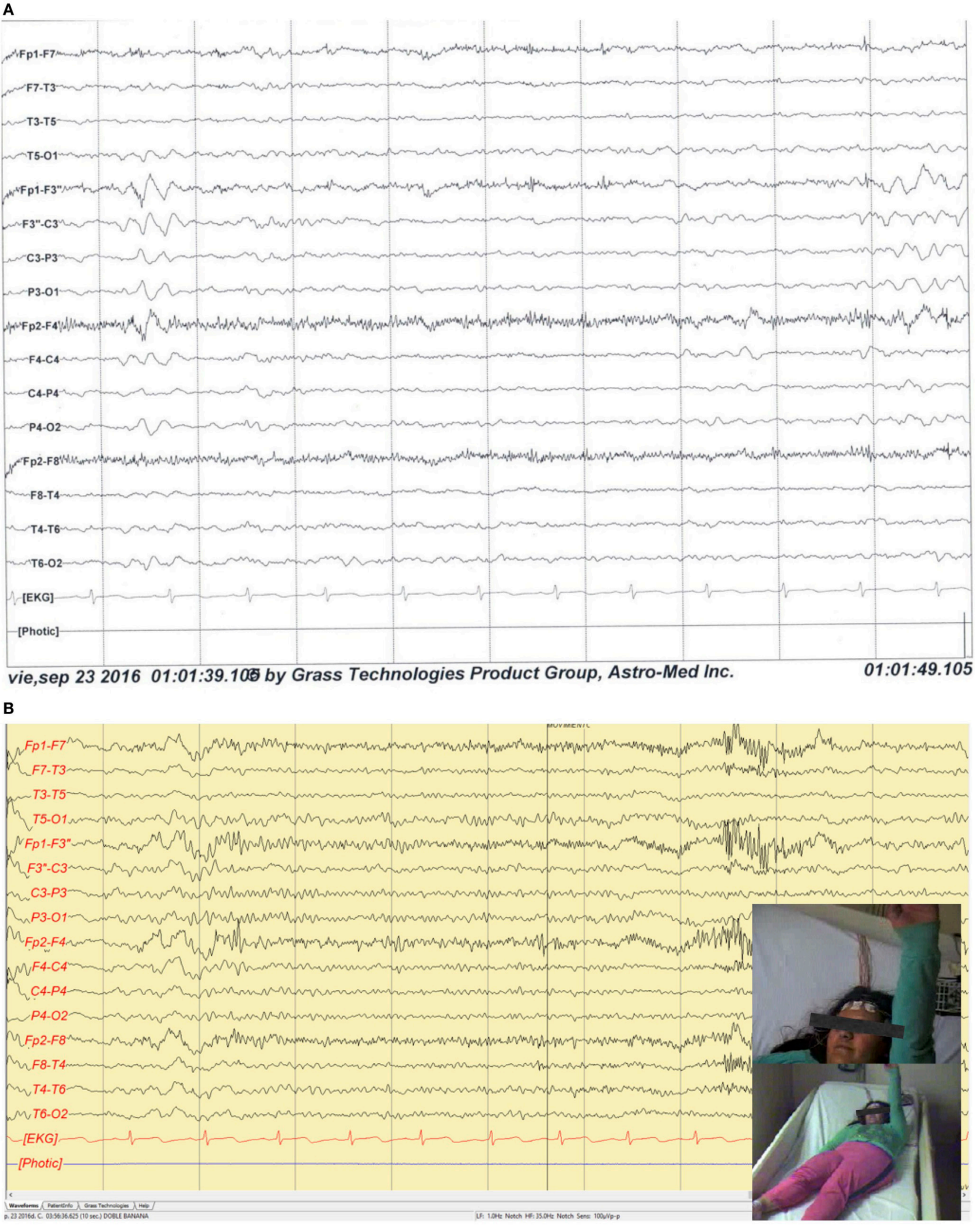
**(A)** Sleep interictal scalp EEG shows epileptiform interictal discharges and focal slowing over the left fronto-central regions. Filters: 0.3–70 Hz. Notch: 60 Hz. Sensitivity: 7 µV/mm. **(B)** Ictal scalp video-EEG recording illustrates a clinical seizure onset over the left frontal central regions characterized by a fast and low voltage pattern with clonic right hemi-body seizures with Jacksonian march without loss of consciousness and preserved language. Filters: 1.0–35 Hz. Notch: 60 Hz. Sensitivity: 100 μVp-p.

The patient’s vital signs, neurological, and psychiatric status were within normal limits. The complete blood count, liver and renal function tests, blood electrolyte levels, and glucose, were all below normal range. Brain 3 T MRI showed a mild cortical dysplasia on the left precentral gyrus; head-PET scan showed a hypometabolic area in the left frontal insular zone. A 10-h video-EEG recording showed 185 ictal (170 electrographic and 15 electro-clinical seizures) events of awake and sleep, mainly during sleep (10/15), characterized by brief focal clonic right hemi-facial seizures with or without Jacksonian march without loss of consciousness and preserved language, and with electrographic onset on the left fronto-central region; interictally showed 7–8 Hz background and 3–4 Hz focal slowing and epileptiform activity on the same region (Figure 2).

The patient was informed about the cathodal tDCS as a noninvasive brain stimulation technique and was asked to participate in this intervention. She agreed to proceed with the intervention and was enrolled after signing the consent form. We performed a pre- and post-cathodal tDCS scalp EEG and evaluated the adverse side effects after each tDCS session. Additionally, the patient reported a 3-month baseline for seizure frequency using a seizure diary and then, subsequently, continued to record her seizure frequency for the follow-up visit. The patient is currently undergoing pre-surgical evaluation for epilepsy surgery.

### Intervention

**Figure 3:**
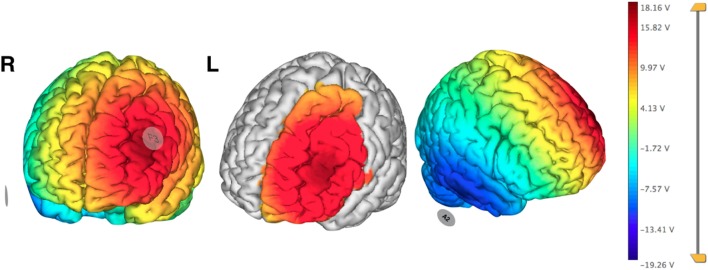
Pre-treatment planning electrical field visualization of cathodal transcranial direct current stimulation protocol over F3 using 2 mA. Anode in contralateral mastoid (A2). Neuroelectrics Instrument Controller software©, Enobio 8. Barcelona, Spain. Abbreviations: R, right; L, left.

Neuronic^®^ (Havana, Cuba) was used for the EEG recording, while a tDCS Stimulator from TCT Research^®^ (Hong Kong, China) was used for therapy. Electrode areas measured 5 cm × 7 cm for anode and 5 cm × 5 cm for cathode. Stimulation was monitored at all times by the researchers. Using EEG visual inspection, we determined the pre-stimulation area to be the most active epileptiform zone, and, therefore, were able to localize the appropriate stimulation area. The cathode of the stimulator was placed on F3 using the 10–20 EEG system and the anode on the contralateral mastoid (Figure 3). Pre- and post-scalp 30-min EEG recordings were analyzed visually and spikes were counted manually on the F3 channel stimulated. She received in total seven sessions of cathodal tDCS (2 mA, 30 min); the first three sessions were applied over three consecutive days, and the remaining four sessions of tDCS were each given at 2-week intervals with a follow-up at 1 year.

### Safety and Follow-up

During the tDCS sessions, the patient only reported mild itching sensation at the beginning and during the intervention. After the first three cathodal tDCS sessions, the patient reported presenting only one epileptic seizure per day. At the end of the seven cathodal tDCS sessions, the patient reported only one focal clonic seizure of her right arm each month. At the 1-year follow-up, the patient reported a single seizure per month, limited to the right upper extremity, related to her menstrual period. Scalp EEGs did not show any qualitative changes in the number of spikes on pre- nor post-tDCS.

## Discussion

We present an adult patient with epilepsy and neuroimaging compatible mild focal frontal cortical dysplasia refractory to AEDs, who improved substantially with several tDCS sessions and long-term follow-up.

Focal cortical dysplasia is a common anomaly during cerebral cortical development, being highly associated with pharmaco-resistant epilepsy, entailing the necessity of resective epilepsy surgery for treatment (9). However, there are alternative treatments for patients who are not candidates due to epileptogenic lesion in eloquent areas, medical conditions, or those who refuse epilepsy surgery (10). Neuromodulation treatments, such as tDCS, a noninvasive method that modulates cortical excitability, has been applied in patients with epilepsy and refractory epilepsy, with heterogeneous etiologies, and with positive results in the majority of the studies published (2, 5).

Fregni et al. (6) steered the first randomized controlled study of the outcomes of tDCS treatment in 19 subjects with intractable epilepsy and malformations of cortical development. Ten patients underwent active treatment (one session; 1 mA, 20 min) by positioning the cathode over the epileptogenic zone previously recognized by EEG, and the anode over an area without epileptic activity. The nine patients, in the placebo group, had the electrodes positioned in the same areas, but had the stimulator turned off after 5 s to produce only a tickling sensation, mimicking the tDCS itching. Epileptic discharges and number of seizures were recorded before the treatment, immediately at the end of the session, and 15 and 30 days afterward. Significant reduction of epileptic discharges (64.3%) was achieved in the tDCS treatment group, as well as a leaning to seizure reduction. The main finding of the research was the absence of prompting or increment in seizures due to cathodal tDCS, and that it is well tolerated by patients (6).

Yook et al. (7), applied cathodal tDCS in an 11-year-old female who had focal cortical dysplasia manifested as congenital bilateral perisylvian syndrome refractory to AEDs. The tDCS cathode was allocated between P4 and T4 in the 10–20 EEG. 2 mA of cathodal tDCS were used for 20 min per day, 5 days a week for 2 weeks. After a period of 2 months at treatment termination, only six seizures ensued. A second session of 2 weeks with same conditions of tDCS treatment was applied. In contrast to the eight seizures per month presented by the patient, only one seizure attack occurred 2 months after the second intervention, making a great improvement (7).

The tDCS’s mechanism of action is not certain, but seems to involve a hyperpolarization and depolarization and modification of synaptic functions (3, 11). The effects of tDCS vary depending on the parameters used, which includes current density, session regularity, time of stimulus, electrode dimension, polarity, and the position of the stimulation electrode (2, 12). For example, Nitsche and Paulus (13) reported that a 20 min session of tDCS in healthy subjects induce sustained cortical excitability for up to 90 min after the end of stimulation (14). However, long-lasting effects of cathodal tDCS in epilepsy are unknown.

Several studies have emphasized the potential of tDCS to improve synaptic plasticity, dependent NMDA receptors in neurological disorders (15, 16). Additionally, the brain-derived neurotrophic factor, the intensity, and frequency of stimulation, are apparently crucial in modulating neuroplasticity (17, 18). The NMDA receptors seem to be indispensable for induction and the preservation of neuroplastic after-effects excitability induced by tDCS (3). Our patient had a similar response to the cathodal tDCS as was reported for Yook et al. (7), using 20 sessions of cathodal tDCS for 5 months. We followed our patient for 1 year and the control of epilepsy was maintained. In comparison, Fregni et al. (6), used only one session of cathodal tDCS and did not find any clinical significant reduction in the frequency of seizures (6).

It’s important to know the possibility that tDCS may be able to control seizures through a placebo effect, and this could partly be explained by an improved response expectancy and also, the use of the tDCS device could give positive expectations (19, 20). Nevertheless, the seizure burden is nonstationary and can fluctuate during long periods of time.

Additional studies are required to define the most adequate stimulation protocols, the mechanism of action, and to establish long-term results.

## Ethics Statement

This clinical trial received approval from the Bioethics and Research Committees of the National Institute of Neurology and Neurosurgery in Mexico City. No. 04/16.

## Author Contributions

DS-J: ideation of case report, head patient’s physician, patient’s treatment description, references revision, and paper writing. KG: medical file data subtraction, references revision, and paper writing. JB: references revision, paper writing, and synthaxis. CS: ideation of case report, patient’s treatment description, references revision, and paper writing.

## Conflict of Interest Statement

The authors declare that the research was conducted in the absence of any commercial or financial relationships that could be construed as a potential conflict of interest.
